# Road and Railway Smart Mobility: A High-Definition Ground Truth Hybrid Dataset

**DOI:** 10.3390/s22103922

**Published:** 2022-05-22

**Authors:** Redouane Khemmar, Antoine Mauri, Camille Dulompont, Jayadeep Gajula, Vincent Vauchey, Madjid Haddad, Rémi Boutteau

**Affiliations:** 1 Normandie University, UNIROUEN, ESIGELEC, IRSEEM, 76000 Rouen, France; antoine.mauri@esigelec.fr (A.M.); camille.dulompont@groupe-esigelec.org (C.D.); jayadeep.gajula@groupe-esigelec.org (J.G.); vincent.vauchey@esigelec.fr (V.V.); 2 SEGULA Technologies, 19 rue d’Arras, 92000 Nanterre, France; madjid.haddad@segula.fr; 3 Normandie University, UNIROUEN, UNILEHAVRE, INSA Rouen, LITIS, 76000 Rouen, France; remi.boutteau@univ-rouen.fr

**Keywords:** dataset annotation, hybrid dataset, multi-modal dataset, mono-modal dataset, 3D bounding boxes, localization, distance estimation, 3D multi-object detection, deep learning, smart mobility

## Abstract

A robust visual understanding of complex urban environments using passive optical sensors is an onerous and essential task for autonomous navigation. The problem is heavily characterized by the quality of the available dataset and the number of instances it includes. Regardless of the benchmark results of perception algorithms, a model would only be reliable and capable of enhanced decision making if the dataset covers the exact domain of the end-use case. For this purpose, in order to improve the level of instances in datasets used for the training and validation of Autonomous Vehicles (AV), Advanced Driver Assistance Systems (ADAS), and autonomous driving, and to reduce the void due to the no-existence of any datasets in the context of railway smart mobility, we introduce our multimodal hybrid dataset called ESRORAD. ESRORAD is comprised of 34 videos, 2.7 k virtual images, and 100 k real images for both road and railway scenes collected in two Normandy towns, Rouen and Le Havre. All the images are annotated with 3D bounding boxes showing at least three different classes of persons, cars, and bicycles. Crucially, our dataset is the first of its kind with uncompromised efforts on being the best in terms of large volume, abundance in annotation, and diversity in scenes. Our escorting study provides an in-depth analysis of the dataset’s characteristics as well as a performance evaluation with various state-of-the-art models trained under other popular datasets, namely, KITTI and NUScenes. Some examples of image annotations and the prediction results of our 3D object detection lightweight algorithms are available in ESRORAD dataset. Finally, the dataset is available online. This repository consists of 52 datasets with their respective annotations performed.

## 1. Introduction

Over the past decades, and especially since 2012, DL has become a very powerful tool in smart mobility due not only to its ability to process large amounts of data but more importantly to provide accurate and relatively fast solutions to various problems in autonomous navigation. The interest in using more and more hidden and intermediate fully connected layers has surpassed traditional techniques in image processing and computer vision, especially in pattern recognition, object detection, and classification. Convolutional Neural Network (CNN) [1] is one of the most popular deep neural networks. Our CNN approaches that use datasets are dedicated to real-time 3D object detection and tracking for both road and railway smart mobility. The following tasks require a dataset for training such as:

1. 2D object detection [2,3,4,5,6,7],
2. Object distance estimation [8,9,10,11,12,13,14],
3. 3D object centers, 3D object detection (center, dimension, and orientation) [2,3,4],
4. Object tracking [2],
5. Semantic segmentation [5,15,16,17].

We also note an abundance of methods for road traffic applications, but a lack of methods for autonomous trains [18,19,20]. The absence of datasets largely explained this absence of methods for railway smart mobility with ground truth data dedicated to 3D object detection, localization, tracking, and semantic segmentation. In this paper, we propose to fill these gaps by proposing our hybrid and multimodal dataset dedicated to road and railway smart mobility. This is a real asset for our research work on environment perception, where the training of DL models on real datasets, with ground truth, is more than necessary to ensure the various tasks of smart mobility such as object detection, distance estimation, tracking, semantic segmentation, scene analysis, and understanding, etc. The main issue for the autonomous vehicle is to obtain data on the surrounding environment with the help of sensors to process the data and adapt its behavior according to this information. A good perception of the environment requires apprehending the following problems:

- Mapping and localization: this represents the establishment of spatial relations between the vehicle and the static surrounding objects. The vehicle must know its position on the map.
- Detection and tracking of moving objects: the vehicle must be able to distinguish between static and dynamic objects, but it must also know its position to them at all times, and therefore follow, or even anticipate, their trajectories.
- The classification of objects (cars, buses, pedestrians, cyclists, etc.).

To enable these actions, there are many sensors related to the perception of the environment dedicated to automated driving. The most frequent are: cameras (mono and stereo vision), Radars, 3D LiDAR, IMU, and GPS. There is a strong complementary in the fields of these main sensors. The choice of these sensors depends on the result that we want to obtain and the problem to be solved. For example, the LiDAR is used in particular because of its high accuracy in measuring the distance of obstacles/depth detection in the outdoor environment [2,3,4,5,6,7]; however, it does not provide information on the class of objects. This is why it is necessary to combine it with another sensor such as a camera. This data fusion will, however, require a calibration and a synchronization between all sensors. With the information on object distances from the camera, it is possible to project it in 3D space by combining this information with the intrinsic characteristics of the camera. By providing in input the depth data associated with the RGB 2D image we can then obtain in output a pro-depth image. All this will allow us to obtain information on the obstacles such as their position, speed, type, width, orientation, etc. and after computer processing, to generate their representations in the form of a bounding box.

The best performing methods based on DL for object detection are generally based on a CNN separated in two modules [1,21,22]. The first one is dedicated to the region proposal, which returns the coordinates of the bonding boxes in which an object is possibly present. The second module then performs detection and returns the class of the object present in the proposed region. Although the detection performance of these methods is excellent, they are computationally intensive and not well suited for real-time applications. The YOLO algorithm [23] proposes a different approach and uses a single CNN to predict the position of the objects and their class while maintaining consistent detection performance.

We present in this paper our new ESRORAD dataset (available for downloading at https://drive.google.com/file/d/1Y4nvMq_yKmmKABeMHp8KvqoiAj0wtV0i/view?usp=sharing accessed on 21 April 2022, GitHub page at https://github.com/rkhemmar/Datasets-and-Annotations.git accessed on 21 April 2022). Firstly, we present our virtual multimodal dataset dedicated to road and railway environments. To develop it, we have used the video game Grand Theft Auto V (GTAV) [3] with realistic virtual scenes. The GTAV dataset is devoted to training CNN algorithms for 3D object detection. It includes images taken from the point of view of both cars (road) and trains (railway). Secondly, we will present our real multimodal dataset collected in both road and railway real environment and that includes 34 videos and 100 k images. To validate our new dataset, we present results obtained through our lightweight real-time 3D object detection [24]. By using a pretrained model on ESRORAD dataset, our approach outperforms state-of-the-art methods. Due to different experiments with our 3D object detection CNN algorithms [3,24], we have validated the new ESRORAD dataset for use in the training and validation of CNN models and algorithms dedicated to autonomous navigation and smart mobility.

The remainder of this paper is organized as follows. Section 1 introduces this paper. In Section 2, we review the related work for both monomodal and multimodal datasets for both road and railway smart mobility. In Section 3, we introduce our virtual GTAV multimodal road and rail dataset. Our new real multimodal road and railway dataset will be presented in Section 4. Dataset annotation process will be presented in Section 5. Experimental results and analysis are presented in Section 6. Finally, the conclusions and future directions are outlined in Section 7.

## 2. Related Work

To ensure effective learning, any deep learning-based approach such as CNN requires a ground truth dataset (also called “ground truth”). One of the main performance factors of any of these approaches in terms of accuracy is the quality and quantity of the data in the dataset. Among the datasets dedicated to autonomous vehicles, the vast majority are for autonomous driving in road environments and focus on object detection and scene segmentation. We can divide them into two main families [3,24,25]: (1) Datasets without ground truth data (images as a single modality), and (2) datasets with ground truth data (as a multimodality). Some of them are mono-modal (e.g., based on camera as single modality), and others are multi-modal (e.g., multisensor fusion system-based camera and LiDAR).

### 2.1. Monomodal Dataset

For training and validation of DL methods, dataset are essential. These ones must contain sufficient ground truth data (position of objects on the image, type of object, 3D position of objects, depth map, etc.) and sufficient images to be used by DL methods. Collecting only RGB images without ground truth is a relatively reasonable task in terms of time and cost. It simply requires one or more inexpensive RGB cameras. This is an advantage when we want to quickly develop datasets for training and CNN inferences. This is still relatively less time consuming and expensive than building the multi-modal dataset often including range data from LiDAR or Radar [3,24].

Various single-mode datasets have been developed in recent years [26,27,28,29], which provide 2D annotations (bounding boxes and masks) and scene segmentation labels for RGB images.

CityScape [27] is a Reference Benchmark and large-scale dataset for training and testing semantic labeling approaches at the pixel and instance level. It also includes various sets of stereo sequences recorded in the streets of 50 different cities. 5000 images with high-quality pixel-level annotations and an additional 20,000 images with coarse annotations to allow methods to exploit large volumes of weakly labeled data [27]. A very recent dataset, DriveSeg [30], developed by MIT and Toyota, shows the value of temporal dynamics information with 12 classes of agents for the driving scene segmentation task. We can also find other datasets dedicated to pedestrian detection annotation such as PascalVOC [31,32,33,34]. INRIA dataset [32], TUD-MotionPairs [33], and NightOwls [34].

### 2.2. Multimodal Dataset

#### 2.2.1. Road Smart Mobility Dataset

Although mono-modal datasets are less costly and time-consuming than multimodal datasets, the latter have a pivotal advantage in supervised learning approaches as ground truths for different types of sensors (LiDAR, RADAR, GPS/IMU, and Cameras) and are available within the same dataset [3,24].

The most popular multimodal road dataset is KITTI [35] (Karlsruhe Institute of Technology and Toyota Technological Institute), which includes rich ground truth from stereo cameras, LiDAR, and a GPS/IMU unit. It is one of the most widely used dataset in the road environment, mainly for tasks such as object detection, distance estimation, and tracking. The video streams recorded throughout 6 h include more than 200,000 annotations of 3D objects captured in different scenarios and traffic conditions in Germany. The recordings were captured and synchronized at 10 Hz using an acquisition system equipped with 4‘high-resolution cameras mounted on the car roofs, a 3D GPS/IMU inertial navigation system (for localization, velocity, and acceleration), and a 3D LiDAR (e.g., Velodyne HDL-64E with 100 k points per frame). The camera is calibrated and synchronized with the Velodyne LiDAR and with the GPS/IMU sensor [35]. KITTI includes 3D object tracking tags for different classes of objects (cars, trucks, streetcars, pedestrians, and cyclists) and serves as a baseline for training and evaluation of stereoscopy, optical flow [36], visual odometry, 2D and 3D object detection [2,3,4,5,6,7], depth estimation [8,9,10,11,12,13,14], and 2D tracking [37,38,39].

NuScenes dataset [40], a new and inspired dataset from KITTI [35], was released in March 2019. [40] It is a large public dataset for autonomous driving use cases that includes 1k driving scenes of each 20 s in length in very dense traffic conditions, recorded from two diverse cities, Boston and Singapore. The full sensor suite includes a LiDAR, 5 RADAR, 6 cameras to provide 360${}^{\circ }$ of coverage, a GPS, and an IMU [40] unit. The dataset includes 1.4M 3D bounding boxes manually annotated for 23 object classes with attributes including visibility, activity, and pose. Additionally, there are 1.1 B LiDAR points manually annotated for 32 classes as well. Compared to KITTI, there are 10× more images in NuScenes with more diverse instances and information useful for training methods for 2D/3D object detection, depth estimation, and tracking [3,24].

In July 2020 NuScenes-LiDARSeg (i.e., LiDAR semantic segmentation) was launched, in which each LiDAR point of the 40k key images in NuScenes with one of the 32 possible semantic labels was annotated. As a result, NuScenes-LiDARSeg contains 1.4 billion annotated points in 40k point clouds and 1k scenes (850 scenes for training and validation, and 150 scenes for testing). The recordings are made in Boston and Singapore, as the two cities are known for their heavy traffic and difficult driving situations. In addition, the routes are chosen to capture as much diversity as possible in terms of vegetation (urban, residential, and natural), time (day and night), buildings, weather conditions, and vehicles, thus enriching the data set. From a large number of training data, 15 h of driving (242 km driven at an average speed of 16 km/h) is selected. To vary the frequency of occurrences of the different classes, scenes containing rare objects (ambulances, animals, etc.) or potentially dangerous situations are included. Taking into account these criteria, 1000 scenes of 20 s each are manually selected [3,24].

The Road event Awareness Dataset for Autonomous Driving (ROAD) [25] includes 22 videos (each 8 min long) with 122 K of annotated frames, and 560 K bounding boxes of sensing with 1.7 M individual labels. ROAD is a dataset that was designed to test the situational awareness capabilities of a robot car. The annotation process follows a multi-label approach in which the road agents (vehicles, pedestrians, etc.), their locations, as well as the actions they perform are identified [3,24,25].

Argoverse 2 [41] is an open-source dataset for autonomous driving data and high-definition maps from six U.S. cities: Austin, Detroit, Miami, Pittsburgh, Palo Alto, and Washington, D.C. Its release builds upon the initial launch of Argoverse 1 [42]. Argoverse is supported by detailed maps for testing and training autonomous vehicles. It includes geometric and semantic metadata, such as lane boundaries and driveable area, that make it possible to develop more accurate perception algorithms, that in turn will enable self-driving vehicles to safely navigate complex city streets. It contains 1k 3D annotated scenarios with LiDAR, stereo imagery, and ring camera imagery and 250k scenarios with trajectory data for many object types. The sensor suite includes all roof-mounted two LiDAR sensors, seven ring cameras, and two front-facing stereo cameras, which are more than that which are used in the KITTI [35] and NuScenes [40] datasets. Apart from the 3D annotations with tracking information for 15 objects of interest [42], it also includes support for motion forecasting, an essential attribute for predicting the location of tracked objects in the near future.

Another multimodal dataset with support for motion prediction is Lyft [43], which uses 40 and 64-beam LiDAR on the roof and bumper and six 360${}^{\circ }$ cameras synchronized with the LiDAR’s to provide the center of each camera’s field of view when images are captured. It includes manually labeled 3D bounding boxes for 4 object classes that provide contextual knowledge through human annotation, an important dominance for scene understanding applications. Waymo [44] is a similar open dataset with coverage in both perception and motion, collected via large-scale, high-quality LiDAR and cameras. It includes 1150 scenes (each 20 s long). The sensors produce annotated data with 2D (camera image) and 3D (LiDAR point cloud) bounding boxes [3,24].

TITAN [45] is a database of 700 raw video clips of 10 to 20 s labeled (with odometry) captured from a moving vehicle in urban traffic scenes in Tokyo. In total, this dataset comprises 10 h of recordings at 60 FPS and 50 tags, all captured with a GoProHero7 camera with an IMU that records synchronized odometry data at 100 Hz. These 700 short raw video clips are then annotated at a sampling rate of 10 Hz. The recordings are made to capture different road scenarios and to define, after learning vehicle states and actions, the pedestrian age groups and pedestrian target action attributes. TITAN is similar to ROAD in the sense that both take into account the actions performed by humans present in the road scene and provide a spatio-temporal location for each person using multiple action labels. However, TITAN is a collection of much shorter videos that last only 10 to 20 s [3,24,45]. CityScapes [27] is a large-scale dataset including stereo video sequences acquired from 50 different cities. Cityscape has high-quality pixel-level annotations of 5 k frames and is intended for the evaluation of semantic urban scene understanding tasks. Cityscapes 3D [46] is a new extension of the original dataset with 3D bounding box annotations for 3D object detection, for example. The Pascal Visual Object Classes (PascalVOC) [31] challenge is not only a dataset but also an annual competition and workshop. The dataset is open access and includes images together with ground truth annotation and evaluation software. PascalVOC aims different challenges for computer vision applications such as pedestrian detection, object classification, segmentation, etc.

Usage of simulators such as CARLA [47] or SYNTHIA [48] for generating synthetic data is very helpful, especially when the available dataset does not offer the required type of instances. They offer simple interfaces that can be used for generating any type of scenario, point of view, weather, and traffic conditions. The advantages of such datasets are that they are simpler to acquire and the images generated can be automatically annotated. However, the primary disadvantage of these simulators is that they have dated graphics that make it very difficult to do domain matching while adopting the trained models to the real-world images. Recent works have proposed the usage of a popular open-world, graphics-oriented video game GTAV [49] for generating more quality images. Richter et al. [49] used a similar approach to acquire a road dataset similar to KITTI [35] and CityScapes [27]. However, despite many available road virtual datasets, to our knowledge, there are no similar state-of-the-art datasets with ground truth to be used for railway smart mobility [3,24].

#### 2.2.2. Railway Smart Mobility Dataset

Although the number of public datasets for road environments has increased in recent years, rail applications are not as well developed. There is a notable lack of work on autonomous trains/trams and this also affects road safety at rail intersections or where road and tramway tracks merge because this environment is not well known [3,24].

To our knowledge, there are only a few image datasets dedicated to smart rail mobility [50,51]. However, these are not dedicated to 3D detection and do not have ground truth information on depth estimation, which limits their use in real smart mobility applications. The RailSem19 [50] is the first published dataset to offer railway data. In the end, more than 8500 scenes, and 350 h of rail and streetcar traffic were collected in 38 different countries and various weather conditions. The data includes over 1 k examples of rail intersections and 1200 images of tramway environments. The limitation of this dataset is that it has no ground truth and therefore no information on the depth of the objects constituting the captured environment. Therefore, these datasets cannot be used for the training or evaluation of 3D object detection approaches [3,24].

Our perception work is mainly based on supervised DL approaches that require a lot of data that is essential for training. However, despite the availability of these data for smart road mobility, we have observed above that no dataset with ground truth is available for the railway domain. Of course, there is a dataset dedicated to railways, RailSem [50], but this one does not have any data on the depth or on the 3D position of objects. To overcome this problem, we decided to develop our own multimodal datasets dedicated to both road and railway smart mobility.

We have therefore developed two datasets: 1. Virtual multimodal dataset based on the video game GTAV [24]. The advantage of these approaches is that they allow a quick and easy acquisition of data, thanks to an automatic annotation process for the labeling of ground truths. However, this comes at the cost of a lower fidelity to reality. 2. Real multimodal dataset based on an acquisition of real road and railway data carried out in two cities of Normandy: Rouen and Le Havre. This is what we will present in the following sections [3,24].

## 3. Virtual Multimodal Road and Railway Dataset

### 3.1. Introduction

The development of our ESRORAD real dataset required 3 years of work (2019–2022). It was therefore not possible to train our CNN algorithms for 3D object detection on railway scenes. To overcome this problem, we started by developing a virtual multimodal dataset devoted to both road and railway smart mobility. We, therefore, used the GTA video game to acquire a railway dataset for 3D object detection. In the literature, some datasets was developped for road environment [52] but without any railway scenes with ground truth data. Our virtual multimodal road and railway dataset was acquired with a data acquisition pipeline from [49]. We used a modified version of the game code to allow the user to drive, for example, a tramway or a train. This allows us to develop a hybrid dataset including both road and railway scenes, which are necessary to train and evaluate our 3D object detection approaches.

### 3.2. Virtual Dataset Architecture

**Figure 1:**
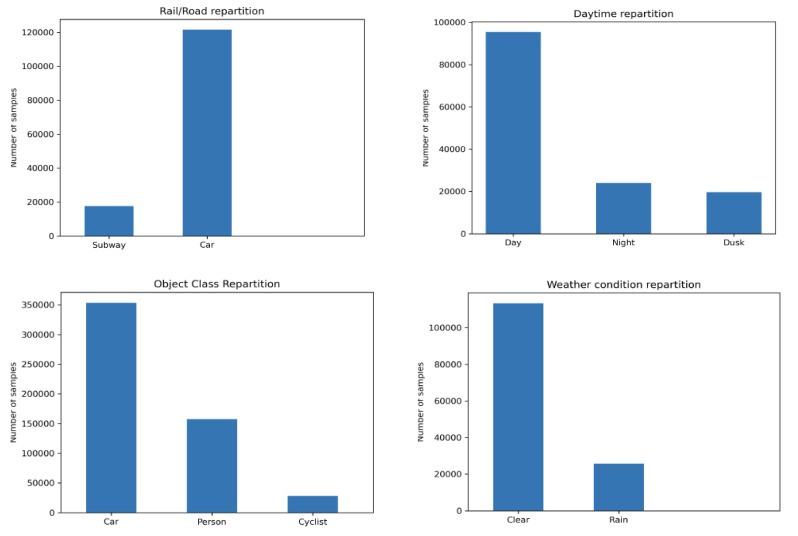
GTAV dataset statistic details: **top left**: road/railway distribution, **top right**: the image daytime distribution, **bottom left**: class distribution, and **bottom right**: weather condition distribution [24].

The ground truth includes the 2D and 3D bounding boxes, the object class, the depth map, and the semantic map. We have do the different annotations by taking into account the point of view of a stereoscopic camera. Some statistics for our dataset can be found in Figure 1.

**Figure 2:**
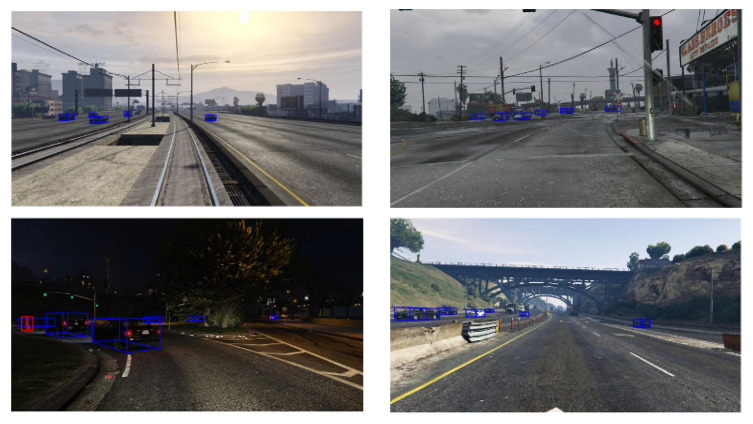
Some examples of our virtual multimodal road and railway GTAV dataset. Images include 3D bounding box ground truths. **Top left**: clear weather railway, **top right**: rainy weather road, **bottom left**: night scene, and **bottom right**: clear weather conditions. The ground truths shown are for the same classes as the KITTI dataset’s classes: Car (blue bounding boxes), Person, and Cycle (red bounding boxes) [24].

For reasons of compatibility of our GTAV dataset with the KITTI 3D object detection dataset, we have chosen to annotate only the classes which are common to both datasets (car, person, and cyclist). Since the first version published in [24], we have improved our GTAV dataset by integrating more annotated images. It currently includes 220 k stereoscopic images (e.g., 110 k samples). Each image sample includes a ground truth data and a depth map for 3D detection. Figure 2 illustrates some examples from our GTAV dataset.

## 4. Real Multimodal Road and Railway Dataset

### 4.1. Introduction

To complete these research works as well as possible and to feed all the information for the autonomous railroad, our objective thus as explained previously, is to create a database of real images, at the same time including images in a road situation, but also with a direct view on tramway rails, in the whole urban environment.

The work thus consists in the realization of a database of real images for the training of DL algorithms for the detection of 3D objects. The chosen approach includes 3 essential steps:

1. The realization of a new multimodal acquisition system composed of a camera and a LiDAR. This phase allows us to calibrate the sensor and to synchronize the camera-LiDAR data.
2. Collection of road and rail data via images acquired with the stereoscopic camera as well as the depth of the objects recorded via the LiDAR. Two cities have been the subject of a data collection protocol: Rouen and Le Havre.
3. The annotation of all the collected images. This allows us to constitute the ground truth and will allow us to validate the depth estimates issued during the protocol evaluation.

### 4.2. Architecture of the Acquisition System

**Figure 3:**
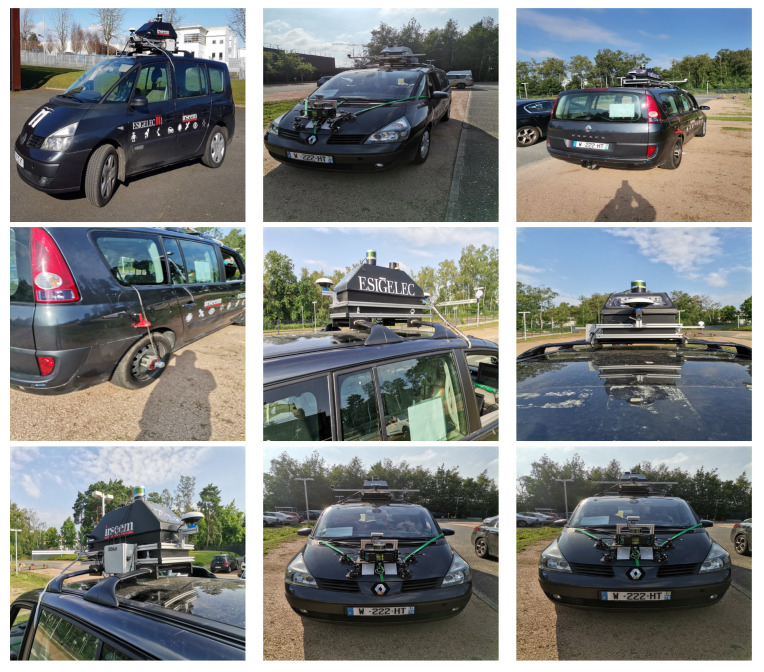
Instrumented IRSEEM Vehicle for data collection in smart mobility. The car trunk on the roof of the vehicle includes the whole sensors (Cameras, GPS/IMU, LiDAR, RADAR, Odometer). The odometer sensor is instrumented on the right rear wheel.

The main difficulties related to this work are due to the required techniques such as the implementation of the acquisition system with the calibration of the sensors or the synchronization between the camera and the LiDAR. To carry out these acquisitions under the most realistic traffic conditions possible, we opted for the use of our test vehicle IRSEEM (e.g., Figure 3), including the following devices:

- Stereoscopic Intel RealSense cameras (L515).
- GPS AsteRx (septentrio).
- Inertial Measurement Unit (IMU) LANDYN (IXblue) composed with an accelerometer, a magnetometer, and a gyroscope. The system also has a post processing software APPS.
- Odometer mounted on the right rear wheel.
- VLP16 LiDAR Velodyne type synchronized with the GPS.
- A real-time data acquisition system RTMAPS (Intempora).

We find, first of all, a GPS that will allow a geolocation of the vehicle in real time. The use of GPS alone has nevertheless some limits, especially when you want a centimetric precision. Indeed, we can find ourselves in the presence of positioning errors (called gps jumps) near buildings or imposing structures; the GPS signal can be, in this case, disturbed during transmission via satellite. To improve geolocation accuracy, we have opted for DGPS (Differential GPS) localization: geo-referenced beacons will send the positioning error back to the vehicle and allow a real-time correction via, in our case, the “Teria” network.

However, an accurate position is not enough for navigation, and information on orientation is also needed, hence the interest in data fusion. For this, the vehicle is equipped with an IMU combining gyroscopes, accelerometers, and magnetometers. The gyroscope allows to measure the orientation and the angular speed. In our case, it is a 3 axis fiber optic gyroscope (FOG), a technology that ensures a very low offset and noise between two GPS positions. A post-processing software is necessary to improve the accuracy by filtering (Kalman filter forward/backward) [39,53] the GPS jumps and anomalies in terms of real-time localization. The fusion of the IMU and the DGPS allows to have all the position and rotation data. The DGPS prevents the drift of the IMU, while the inertial unit prevents GPS jumps.

**Figure 4:**
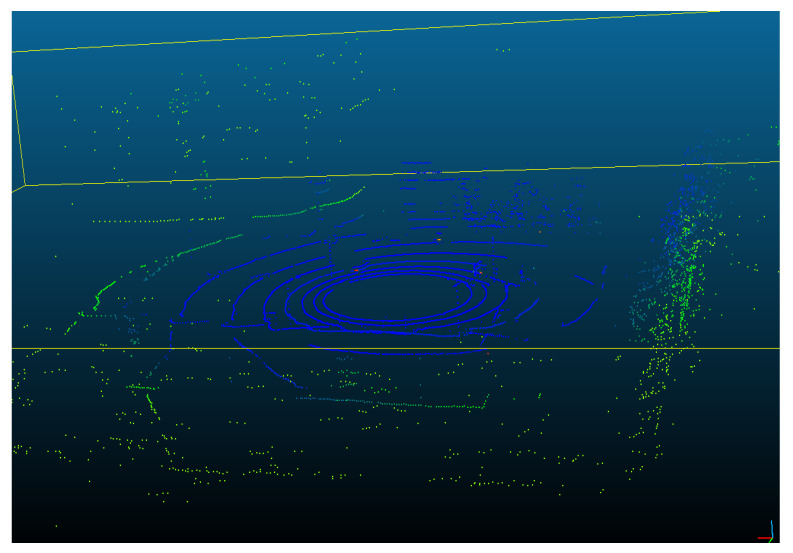
An example of 3D cloud points carried out through Velodyne VLP16 LiDAR. The coloring is related to the distance (depth) of the point from the sensor.

The Velodyne LiDAR VLP16 multi-sheet sensor allows us to obtain a distance map on 16 sheets simultaneously. Each distance measurement is obtained at a fixed angular distance from the previous measurement. It is therefore possible to convert the distance map into a set of 3D points, which is called a point cloud (e.g., Figure 4).

In addition to the LiDAR data, the acquisition system described above also includes a stereoscopic camera to collect RGB images of the road and rail scene. The objective will then be to synchronize each LIDAR scan with the camera image capture corresponding to the same acquisition time and then to project the recorded LiDAR point cloud onto the image. The RealSense L515 cameras have a better RGB resolution outdoors and can measure the distance of each image pixel via their integrated LiDAR calibrated and synchronized.

### 4.3. Calibration and Synchronization of the Acquisition System

#### 4.3.1. Sensors Calibration

The camera calibration operation corresponds to determining the relationship between the spatial coordinates of a point in space with the associated point in the image captured by the camera. We use the pinhole model, which models a camera by a perspective projection by transforming a 3D point in space into an image point. This can be broken down into three successive elementary transformations (e.g., Equations (1) and (2)).

Step 1 of the calibration represents the transformation between the world reference frame and the camera reference frame (whose origin is located at the optical center of the camera). This transformation can be broken down into a rotation *R* and a translation *T* forming the extrinsic parameters of the camera. The transformations 2 and 3 allow it to pass from the coordinates in the camera frame to the image coordinates and thus depend on the intrinsic parameters. All these parameters are necessary to project the LiDAR points onto the recorded camera image. It is therefore necessary to bring together the intrinsic and extrinsic parameters representing the passage from the camera frame to LiDAR (vehicle). The intrinsic parameters are determined by using a calibration chart made of black and white checkerboards.

**Figure 5:**
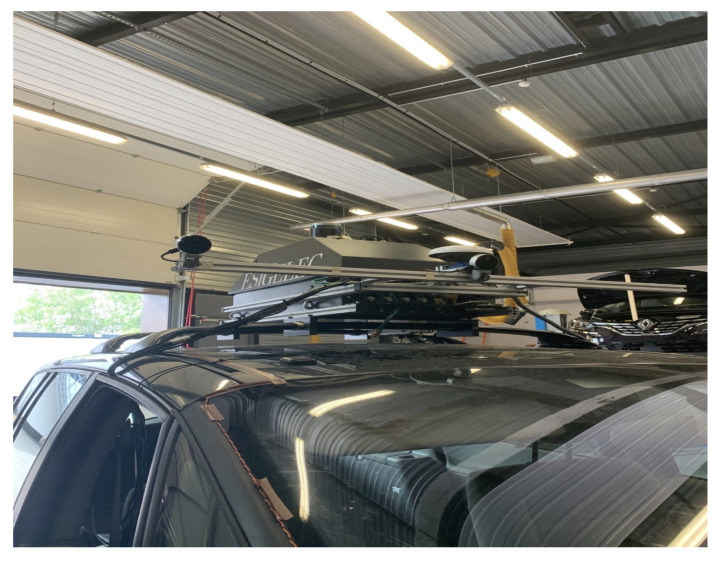
The instrumentation of the camera on the roof of the vehicle. The camera is fixed on a rail just in front of the LiDAR.

The cameras are calibrated, using a checkerboard pattern to determine the intrinsic and distortion parameters of the camera. The extrinsic parameters are determined using the LiDAR function of the camera. To obtain the passage matrix, the camera is instrumented in the trunk on the roof of the vehicle, as shown in Figure 5. The objective is to overlay the LiDAR scans of the vehicle with the LiDAR scans of the L515 camera to obtain the extrinsic parameter matrix. A cube-shaped calibration target is placed in front of the camera and the LiDAR sensors. It consists of three orthogonal planes with known patterns. Once the patterns are detected, the transformation matrix is calculated from the camera to the LiDAR by aligning the planes of the calibration target. Subsequently, it is then possible to determine the transformation from the camera to the image and the resulting extrinsic parameters.

**Figure 6:**
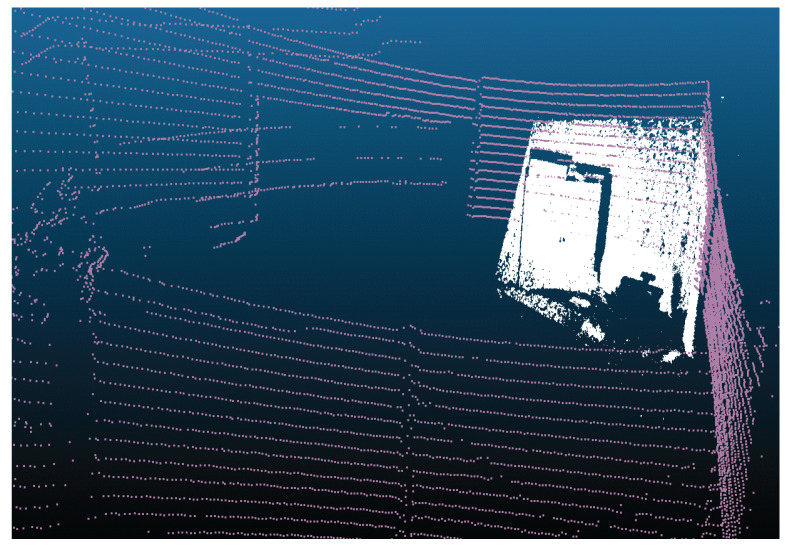
Alignment of cloud points LiDAR of RealSense L515 camera (white) to the cloud point of the Vehicle LiDAR (purple).

To achieve proper intermodal data alignment between the LiDAR and stereo cameras, a camera’s exposure is triggered when the upper LiDAR scans the center of the camera’s field of view. The image timestamp represents the exposure trigger time, and the LiDAR scan timestamp represents the time at which a full rotation of the current LiDAR image is reached. Since the camera exposure time is almost instantaneous, this method generally gives a good data alignment (e.g., Figure 6). Cameras operate at 12 Hz while LiDAR operates at 20 Hz. A LiDAR scan must then be matched to an image by identifying the capture times.

#### 4.3.2. Projection of LiDAR Points on the 2D Image

**Figure 7:**
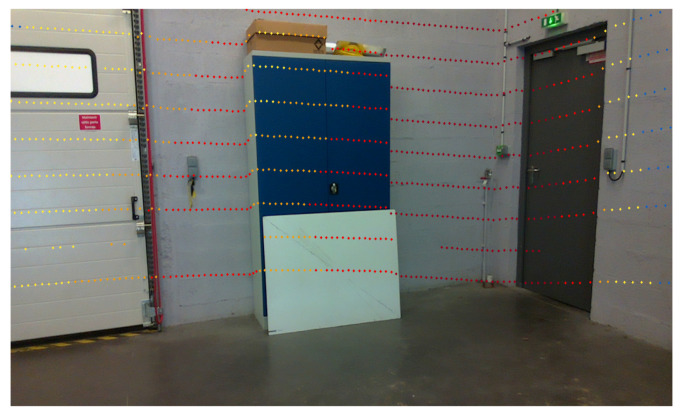
Result of the projection of the LiDAR points on the static image. The coloring is related to the distance (depth) of the point from the sensor.

To verify that the resulting pass matrices are correct, the transformation matrix is applied to the images and point clouds. This allows us to display each LiDAR point on the image. The extrinsic matrix is presented in Equation (1), where the LiDAR points in the camera frame are expressed according to the extrinsic parameters matrix (LiDAR to camera transition) and the LiDAR points. Firstly, we recover the LiDAR points file that coordinates are expressed in the LiDAR reference frame, then we multiply this matrix by the extrinsic parameters matrix composed of the rotation and translation parameters to place the LiDAR points in the camera reference frame (Equation (1)) [54,55].

(1) $$\left|\begin{array}{c} X_{C11}\cdots \cdots X_{C1n}\\ Y_{C21}\cdots \cdots Y_{C2n}\\ Z_{C31}\cdots \cdots Z_{C3n}\\ 1\cdots \cdots \cdots 1 \end{array}\right|=\left|\begin{array}{cccc} r_{11} & r_{12} & r_{13} & t_{x}\\ r_{21} & r_{22} & r_{23} & t_{y}\\ r_{31} & r_{32} & r_{33} & t_{z}\\ 0 & 0 & 0 & 1 \end{array}\right|\left|\begin{array}{c} X_{L11}\cdots \cdots X_{L1n}\\ Y_{L21}\cdots \cdots Y_{L2n}\\ Z_{L31}\cdots \cdots Z_{L3n}\\ 1\cdots \cdots \cdots 1 \end{array}\right|$$

where $r_{ij}$ is rotation verctor *R*, $t_{k}$ is translation verctor *T*, and (x,y) are measured in mm. Secondly, the matrix of LiDAR points expressed in the camera frame obtained in Equation (1)) is used with the matrix of intrinsic parameters of the camera (camera-to-image pixel transition) to compute the pixel coordinates of the image, as it is presented in Equation (2)) [54,55].

(2) $$\left|\begin{array}{c} U_{1}\cdots \\ V_{1}\cdots \\ 1\cdots 1 \end{array}\right|=\left|\begin{array}{ccc} f_{x} & 0 & c_{x}\\ 0 & f_{y} & c_{y}\\ 0 & 0 & 1 \end{array}\right|\left|\begin{array}{c} X_{C11}\cdots \cdots X_{C1n}\\ Y_{C21}\cdots \cdots Y_{C2n}\\ Z_{C31}\cdots \cdots Z_{C3n}\\ 1\cdots \cdots \cdots 1 \end{array}\right|$$

where (u,v) are measured in pixel, $\left(u_{0},v_{0}\right)$ are the pixel coordinates of the optical axis projection, and $\left(f_{k},c_{k}\right)$ are the intrinsic parameters of the camera. We then take the points expressed in the camera frame and multiply them by the matrix of intrinsic parameters to obtain the LiDAR points in the image frame expressed in pixel coordinates. The Figure 7 above illustrates the consistency of tracking curves of LiDAR points in the corners of the wall or cabinet. Later, a color gradient will be added to visualize the depth.

#### 4.3.3. Camera-LiDAR Synchronization

**Figure 8:**
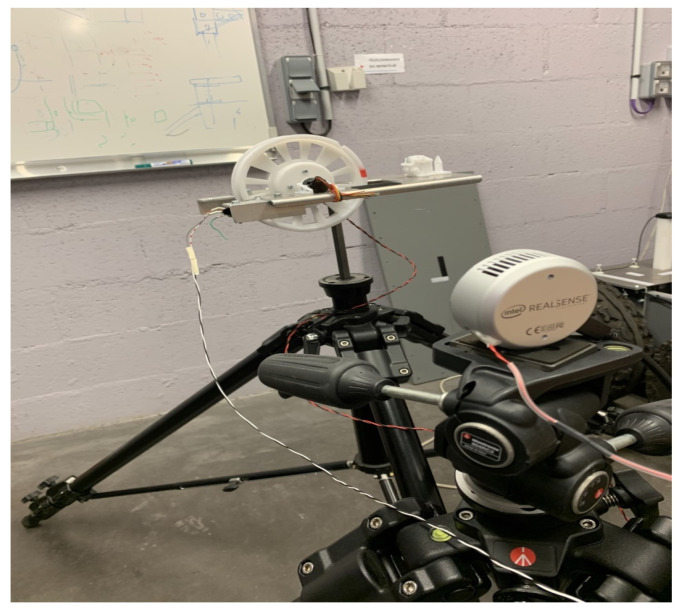
Protocol of validation of the synchronization of the camera and LiDAR data.

During the previous calibration part, the projection, in static, of the LiDAR points on the 2D image did not pose any problem, as the vehicle was at a standstill; thus, even if the LiDAR and the camera operate at different triggering frequencies, the scene remains the same. In dynamics, this shift of triggering is more problematic. Indeed, to perform the projection on moving scenes, it is necessary to associate each LiDAR scan with the corresponding image that was captured at the same time. We, therefore, developed a validation protocol to verify the synchronization of the camera and LiDAR data as shown in Figure 8.

**Figure 9:**
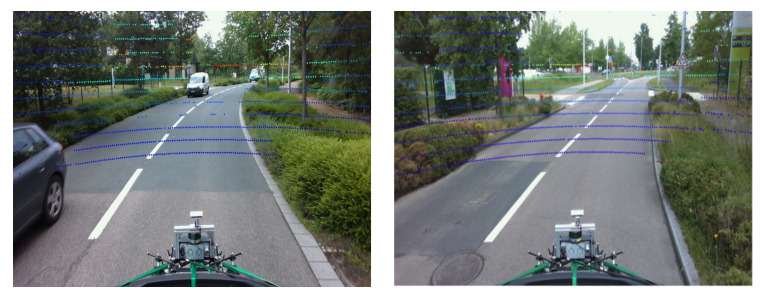
Results of the projection of LiDAR points on the dynamic image. The coloring is related to the distance (depth) of the point from the sensor.

Figure 9 illustrates the coherence of the depth materialized by the gradient aspect of the colors (blue for close objects and red for distant objects) as well as the contours adopted by the LiDAR points for certain objects such as the tree or the vehicle. It is possible that reflections on the bodywork create spurious points.

### 4.4. Data Collection

#### 4.4.1. Route Selection for Data Collection

**Figure 10:**
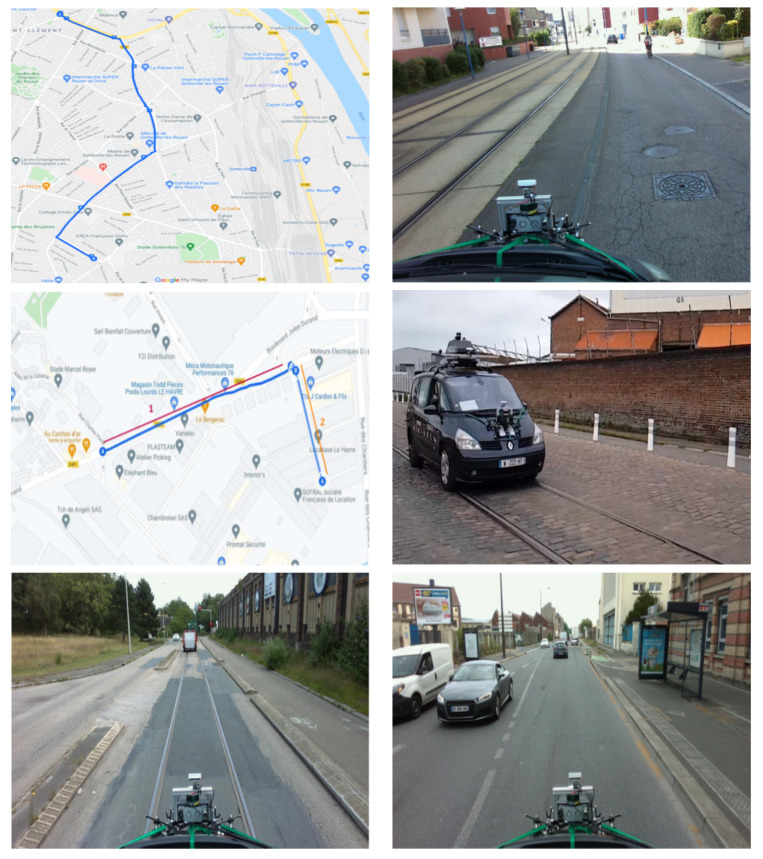
Data collection on both road and railway environment. **Top level left**: route Google Maps Rouen, **top level right**: data collection in Rouen city center, **middle level left**: route Google Maps Le Havre, **middle level right**: data collection in Le Havre city center, **bottom level left**: data collection from railway point of view in Le Havre, and **bottom level right**: data collection from road point of view in Le Havre (France).

To carry out the data collection, we planned a recording protocol, taking into account two Norman agglomerations: Rouen and Le Havre. They have an important road and rail traffic network including not only roads and tramways, but also areas where car traffic is allowed on the railroad. This allowed us to acquire road and/or rail scenes with a direct view of the tracks. This also allowed us to collect data under normal conditions without interruptions of the tramway traffic. Figure 10 shows an example of a scene where the test vehicle is recording data in a multimodal road/railway environment.

Our dataset includes various recorded scenes: road only, railway only (tramway or train), or hybrid road and railway. We were able to collect about 100 k images distributed between Le Havre city (45 k images) and Rouen city (55 k images). The agglomeration of Rouen and Le Havre are a propitious environment for collecting images from both a road and railway point of view due to the overlapping of the tramway tracks and the road in certain cities, which allowed us a recording with a direct point of view on the railways. This allowed us to move with the vehicle in normal traffic conditions and acquire the desired data without interruptions by tramway traffic. We, therefore, selected several precise routes in Google Maps for the acquisitions in the two cities by gathering the maximum criteria (road scenes, railway scenes, hybrid road and railway scenes, possibility to drive on the tramway line, height of the vehicle in case of passage under bridges, etc.). The data collection took place in two steps: 1. Data collection as a validation of the protocol and the chosen routes (between May and July 2022), 2. Real data collection (October 2022) was carried out for one week in the two Normandy cities: Rouen and Le Havre. The data collection was conducted in both morning (9 to 12 a.m.) and afternoon (2 to p.m.) to take into account different significant parameters such as the lighting conditions, weather conditions (rainy, cloudy, and sunny), road traffic, etc.

#### 4.4.2. Data Recording Protocol

Before any data collection, an initialization of the acquisition system is necessary and is conducted in two modes: (1) Static (stop of the vehicle): the IMU based on the FOG is sensitive enough to measure the rotation of the earth and thus be able to give a heading in static. A waiting period of 10 min is necessary to initialize the system. (2) Dynamic (the vehicle in motion): the idea is to move with the vehicle for a few minutes to make the Extended Kalman Filter (EKF) [39] converge by reducing the heading error. Then, the data recording can start and this initialization step is repeated at the end of the recording process as well.

## 5. Dataset Annotation Process

### 5.1. Dataset Annotation Softwares

**Figure 11:**
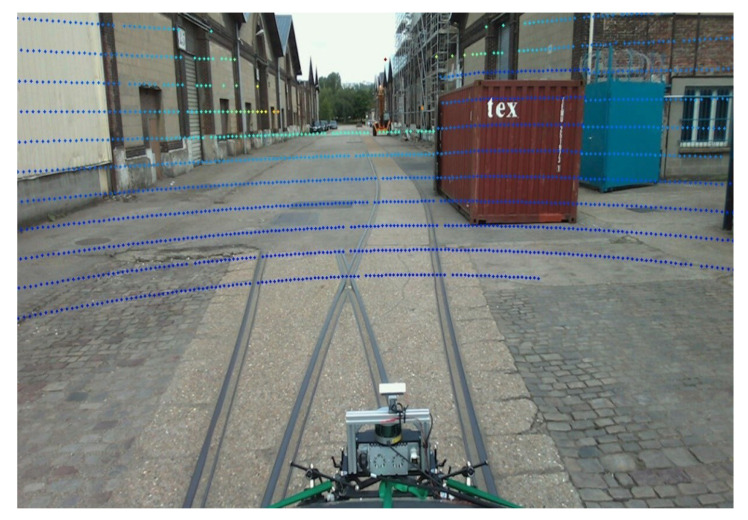
Overview of the result of the projection of the LiDAR points on an image of the railway dataset collected in Le Havre city (France). The coloring is related to the distance (depth) of the point from the sensor.

After many days of data collection, the LiDAR data are exported and then a reprojection is run on the different acquisitions (e.g., Figure 11).

Once the projection is validated, the post-processing part begins, i.e., the documentation of the images with the ground truth. The objective was for each image, to identify in the environment our three main classes (vehicle, pedestrian, cyclist) and also the distance of each object to the vehicle. All this information will constitute the ground truth of the real dataset that will be dedicated to the training and testing of both 2D and 3D object detection algorithms. We have therefore carried out a Benchmarking of all the annotation tools that exist in the literature. The idea is to display the LiDAR point clouds, to manually place 3D boxes corresponding to the class of the identified object, and then to display the result on the color image. This labeling step will be the last step of the dataset development.

Supervisely and Scale: Regarding our dataset 3D annotation Benchmarking, we have tested different tools such as Self-hosted and cloud-hosted tools to annotate comprehensive 3D scenes from LiDAR or RADAR sensors (Supervisely) [56] and Scale [57]. This software was good and reliable but we needed software that is open source and cross-platform compatible as we want to make it possible for everyone to have access to the annotation software.

BAT 3D: A Semi-Automatic, Web-based 3D Annotation Toolbox for Full-Surround, Multi-Modal Data Streams called “3D Bounding Box Annotation Tool” (BAT 3D) [58] that is a novel open access annotation system devoted to a 2D and 3D dataset annotation. It is efficient, accurate, and includes tools for 3D object localization and their dynamic movement using full-surround multi-modal data streams [58]. It is open-source and cross-platform compatible for the 3D annotation. It also consists of many features that the other 3D annotation tool fails to offer. BAT 3D satisfied all of our requirements for the annotation of our dataset. It also consists of many features that the other 3D annotation tool fails to offer.

**Figure 12:**
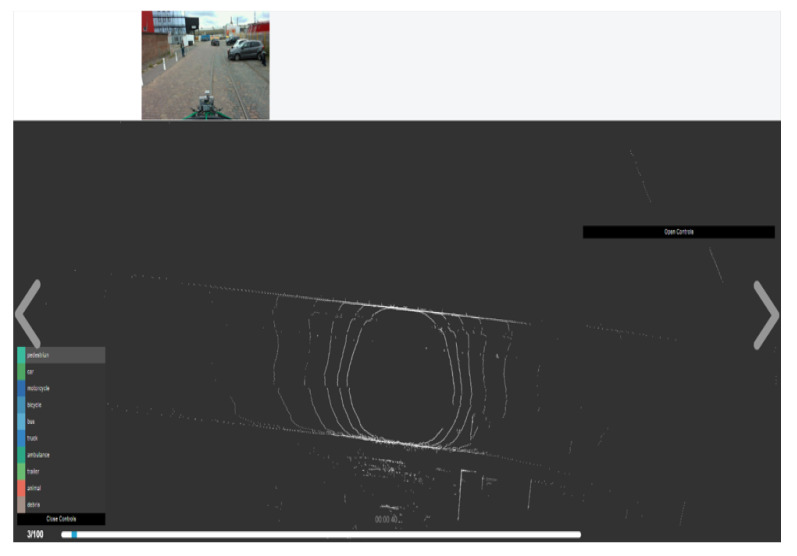
BAT 3D interface with one front image view tacked from our dataset in Le Havre City.

The tool focuses on the challenging task of 2D image and 3D point cloud annotation. The user can annotate scenes directly in 3D and transfer those annotations back into the image domain. Laser data is first annotated with rough bounding primitives, and then a geometric model is used to transfer these labels into the image space. All the six camera images are displayed, but we just needed the Camera front view as we have only made use of one camera for the data collection as shown in Figure 12.

### 5.2. Road and Railway Dataset Annotation

**Figure 13:**
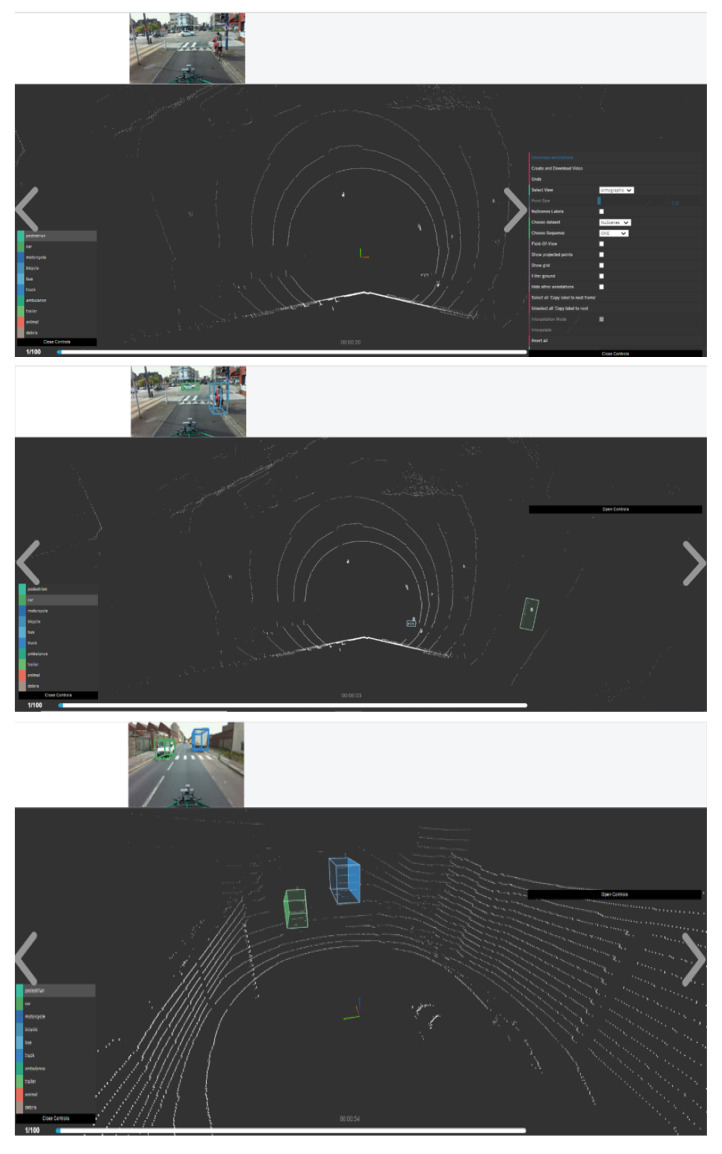
Some annotations examples in ESRORAD dataset. **Top line**: BAT 3D tool view before annotation, **middle** and **bottom up line**: BAT 3D tool view after annotation.

We can draw a bounding box by dragging the mouse along the point cloud projection of the object. The dimensions of the bounding box, such as the length, width, yaw, etc., can be changed. The annotation can be checked from all angles and even from the ground level. We can also interpolate the annotations that are performed; we can copy the annotation performed in one frame to another easily with the interpolation mode and we can change the rotation and translation of the bounding box too. This option can also be used to annotate faster. In the end, we can download all the annotations in JavaScript Object Notation (JSON) format. Figure 13 shows some examples of annotations.

**Figure 14:**
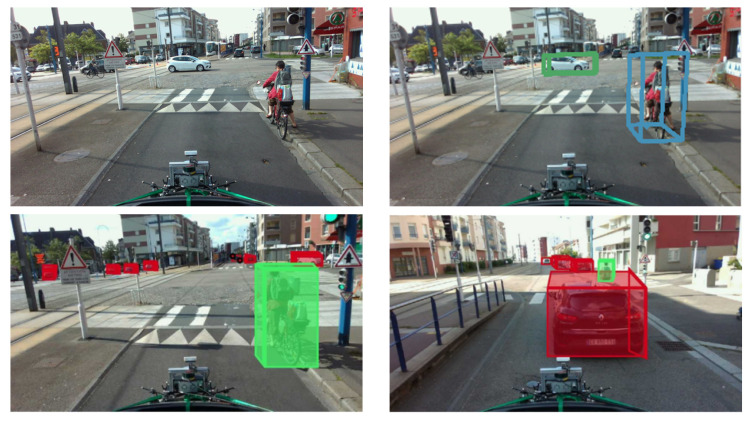
Examples of images annotations in Rouen city center (France). **Top level left**: regular image without any annotations, **top level right**: image with annotations for two classes: car and cyclist, acquired in Rouen city center. **Bottom up level left**: 3D bounding box for two classes: car (in red color) and cyclist (green color), **bottom up level right**: 3D bounding box for two classes: car (in red color) and cyclist (green color).

The results of our annotation through BAT 3D are very clear with the corresponding images and their annotations. The annotations are performed considering mainly with the LiDAR points, but the images are meant as a reference for correction. Figure 14 shows an example of our annotations including 3D bounding boxes drawn on the objects, respectively.

## 6. Experimental Results and Analysis

**Figure 15:**
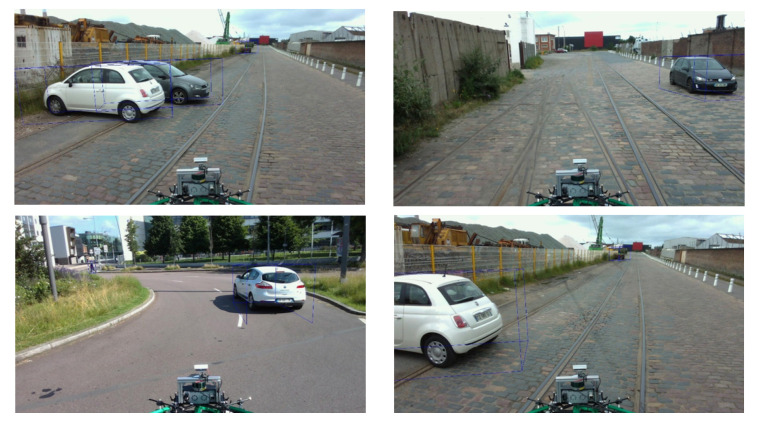
Examples of annotations and predictions performed on the ESRORAD real dataset. **Top left**: image without annotation, **top right**: image with annotation, **bottom two images**: predictions of our object detection algorithm trained on the GTAV virtual dataset.

**Figure 16:**
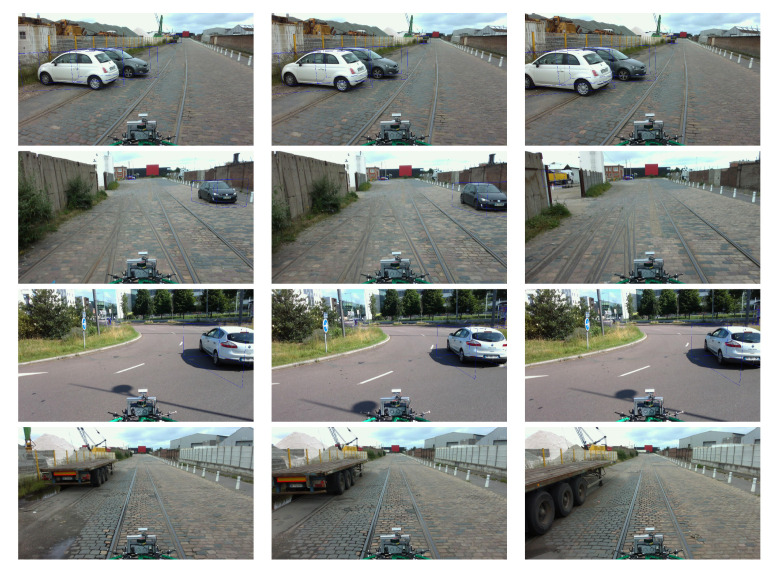
Examples of image annotations and predictions in the ESRORAD dataset. Results obtained through our lightweight 3D object detection algorithm [24] and trained for 300 epochs on the Nuscenes [40] dataset.

The annotation of the images consisted in documenting them with the ground truth. The objective was to identify the different objects in the environment (vehicle, pedestrian, and cyclist) as well as their distance from the vehicle. We used the BAT 3D tool that we modified for the annotation of the images. The annotation consisted in displaying the LiDAR point clouds and manually placing 3D boxes corresponding to the class of the identified object, then displaying the result on the color/RGB image. Figure 15 and Figure 16 illustrates not only some examples of image annotations but also the prediction results of our lightweight 3D object detection algorithm trained for 300 epochs on the Nuscenes [40] dataset.

Why does ESRORAD represent a novelty compared to the state-of-the-art datasets?

- ESRORAD is currently the only dataset, to our knowledge, dedicated not only to road smart mobility (autonomous car) but also to railway smart mobility (autonomous train).
- For the railway domain, there is only one dataset available in the literature
  (RailSem19 [50]), but it does not include ground truth data and therefore it does not allow one to qualify 3D object detection deep learning algorithms or distance estimation approaches.
- ESRORAD is the only dataset that combines road (80%) and rail (20%) scenes with virtual (220K images) and real (100k images) data.
- In the medium term:
  - ESRORAD aims to extend railway data outside cities to integrate rural landscapes such as in the case of long-distance trains.
  - Images and video quality will be improved through a new high-resolution LiDAR. This will allows us to make available high-quality HD datasets for more accuracy and safety.

## 7. Conclusions

In this paper, we presented our new Esigelec engineering high school and Segula technologies ROad and RAilway Dataset (ESRORAD). Our hybrid multimodal dataset is a large scale, first of its kind dataset, that can be institutively utilised for training and validation of models in both road and railway smart mobility applications. The hybrid nature of the dataset is achieved by including synthetic sequences developed with GTAV as a simulator and fully synchronised real images with 3D point clouds acquired using our Instrumented IRSEEM vehicle dedicated for data acquisition. In terms of quantity, our dataset includes 34 videos, 2.7 k virtual images, and 100 k real images collected in two Normandy cities, Rouen and Le Havre. The complete dataset is acquired in several fields of view in numerous road and railway traffic conditions. The images are annotated with 3D bounding boxes showing at least three different classes of objects, namely, persons, cars, and bicycles. We also provide open access to our dataset to allow researchers and companies to conduct their research works. Additionally, ESRORAD was validated with promising results, while benchmarking was conducted using our different CNN models that were trained under KITTI and NUScenes datasets. Hence, our dataset is an excellent choice for training and validation of different perception algorithms performing tasks such as 3D object detection, object tracking, semantic segmentation, scene parsing, and understanding. However, ESRORAD is extensible to many other challenging tasks. Thus, we are currently preparing other quantitative data that could enrich our dataset. Therefore, we are also committing to collect more road and railway data incorporating more scenes and videos in different weather conditions (rain, snow, sun, clouds, etc.) and different lighting conditions (day and night). In future work, we will compare the performance of our ESRORAD dataset under new evaluation metrics used by more baseline algorithms in the field of 3D object detection for autonomous vehicles (such as our lightweight 3D object detection algorithm trained by Nuscenes and the KITTI dataset). This is part of the next step, and the new version of our dataset, called ESRORAD2, will be available by the end of 2022. There is an urgent need to make available a multimodal dataset, because the majority of large cities include road and railway (tramway) scenes, yet all the research work today focuses only on on-road scenes, as is the case for the autonomous car. 

## Abbreviations

The following abbreviations are used in this manuscript:

ESRORAD	Esigelec engineering high school and Segula technologies ROad and RAilway Dataset
ADAS	Advanced Driver Assistance System
ANL	Autonomous Navigation Laboratory
DL	Deep Learning
RGB	Red, Green, and Blue
GTAV	Grand Theft Auto V
CNN	Convolutional Neural Network
YOLOv3	You Only Look Once
LiDAR	Light Detection And Ranging
LiDARSeg	LiDAR semantic Segmentation)
ROAD	ROad event Awareness Dataset for autonomous driving
RADAR	Radio Detection And Ranging
KITTI	Karlsruhe Institute of Technology and Toyota technological Institute at chicago
RailSem19	A Dataset for Semantic Rail Scene Understanding
FPS	Frame Per Seconde
EKF	Extended Kalman Filter
NUScenes	NuTonomy Scenes
INRIA	Institut National de Recherche en Sciences et Technologies du Numérique
